# Projecting Future Heat-Related Mortality under Climate Change Scenarios: A Systematic Review

**DOI:** 10.1289/ehp.1103456

**Published:** 2011-08-04

**Authors:** Cunrui Huang, Adrian Gerard Barnett, Xiaoming Wang, Pavla Vaneckova, Gerard FitzGerald, Shilu Tong

**Affiliations:** 1 School of Public Health and Institute of Health and Biomedical Innovation, Queensland University of Technology, Brisbane, Australia; 2 CSIRO Climate Adaptation Flagship and CSIRO Ecosystem Sciences, Commonwealth Scientific and Industrial Research Organisation, Melbourne, Australia

**Keywords:** climate change, heat wave, mortality, projection, public health, scenario

## Abstract

Background: Heat-related mortality is a matter of great public health concern, especially in the light of climate change. Although many studies have found associations between high temperatures and mortality, more research is needed to project the future impacts of climate change on heat-related mortality.

Objectives: We conducted a systematic review of research and methods for projecting future heat-related mortality under climate change scenarios.

Data sources and extraction: A literature search was conducted in August 2010, using the electronic databases PubMed, Scopus, ScienceDirect, ProQuest, and Web of Science. The search was limited to peer-reviewed journal articles published in English from January 1980 through July 2010.

Data synthesis: Fourteen studies fulfilled the inclusion criteria. Most projections showed that climate change would result in a substantial increase in heat-related mortality. Projecting heat-related mortality requires understanding historical temperature–mortality relationships and considering the future changes in climate, population, and acclimatization. Further research is needed to provide a stronger theoretical framework for projections, including a better understanding of socioeconomic development, adaptation strategies, land-use patterns, air pollution, and mortality displacement.

Conclusions: Scenario-based projection research will meaningfully contribute to assessing and managing the potential impacts of climate change on heat-related mortality.

Exposure to extreme heat has been associated with both increased mortality and morbidity. A number of epidemiological studies have examined high temperatures in relation to total nonaccidental deaths (McMichael et al. 2008; Stafoggia et al. 2006), to cause-specific mortality (Barnett 2007; Hertel et al. 2009), and to other health outcomes such as emergency department visits and hospitalizations (Knowlton et al. 2009; Wang et al. 2009). In fact, heat waves are the biggest cause of weather-related fatalities in many cities, responsible for more deaths annually than any other form of extreme weather [Luber and McGeehin 2008; World Health Organization (WHO) 2009a].

Recently, heat-related mortality has become a matter of growing public health concern, especially because of climate change (Kovats and Hajat 2008; O’Neill and Ebi 2009). The Intergovernmental Panel on Climate Change (IPCC) indicates that hot weather is likely to increase future heat-related mortality (IPCC 2007a). Urban areas, home to more than half of the world’s population (United Nations 2009), can be particularly vulnerable to heat because of high concentrations of susceptible people (Hajat et al. 2007), the urban heat island effect (Smargiassi et al. 2009), poor urban design and planning (Stone et al. 2010), and the interaction between air pollution and heat (Ren et al. 2006).

People with cardiovascular or respiratory disease, diabetes, chronic mental disorders, or other preexisting medical conditions are at greater risk from heat exposure (Kovats and Hajat 2008; WHO 2009a). The effects of heat are particularly strong in the elderly (Basu and Ostro 2008; Vaneckova et al. 2008a). Other factors that influence the risks of heat-related mortality include social isolation (Naughton et al. 2002; Semenza et al. 1996), low income (Kaiser et al. 2001), low education (O’Neill et al. 2003), poor housing (Vandentorren et al. 2006), lower access to air conditioning (O’Neill et al. 2005), and less availability of health care services (WHO 2009a).

Many study designs have been used to examine the effects of temperature on mortality, including descriptive (Reid et al. 2009), case–control (Naughton et al. 2002), case-only (Schwartz 2005), case-crossover (Smargiassi et al. 2009; Stafoggia et al. 2006), time-series (Hajat et al. 2002; Kan et al. 2007), spatial (Vaneckova et al. 2010), and synoptic analyses (Vaneckova et al. 2008b). Generally, time-series and case-crossover are considered more efficient designs for examining the temperature–mortality relationships in single or multiple locations over time (Basu and Samet 2002; Basu et al. 2005). These designs aim to investigate the health effects of temperature after controlling for potential confounders such as trends and seasonal cycles in mortality and, in some cases, humidity and air pollution (Kovats and Hajat 2008).

The health effects of heat can be estimated using the heat threshold and the heat slope. The temperature–mortality relationship is usually a nonlinear U-, V-, or J-shape. Many studies have quantified cold and heat effects separately, assuming a linear response below and above a threshold temperature (Baccini et al. 2008; Hajat and Kosatsky 2010; McMichael et al. 2008). The heat threshold is the temperature at which the harmful effect of heat begins to occur, and the heat slope measures the size of this effect (Hajat and Kosatsky 2010). A significant geographic variability has been observed in both heat thresholds and slopes. Heat thresholds tend to be higher in warmer locations, suggesting acclimatization (Baccini et al. 2008; Medina-Ramon and Schwartz 2007).

Many studies have found associations between high temperatures and mortality, but more research is needed on the impacts of climate change on future heat-related mortality (Campbell-Lendrum and Woodruff 2007; Costello et al. 2009; Ebi and Gamble 2005; Huang et al. 2011; WHO 2009b). Scenario-based projections have been used as a key approach for policy making and planning in the context of uncertain future conditions (Varum and Melo 2010). The IPCC has developed a set of scenarios in its *Special Report on Emissions Scenarios* (SRES; Nakicenovic et al. 2000). These scenarios are not assigned probabilities but, rather, can be deemed as possible futures, which depend on demographic, technological, political, social, and economic developments (Nakicenovic et al. 2000). Scenarios are used not to better predict the future but to better understand uncertainties in order to reach decisions that are robust under a range of possible futures (Moss et al. 2010). Informed by quantitative or qualitative evidence, projections provide decision makers with information on a variety of future trends, contexts, risks, and opportunities. Projecting heat-related mortality under climate change scenarios will therefore help decision makers in planning adaptation strategies and communicating the future health risks of climate change to the public and politicians (Menne and Ebi 2006).

Understanding and managing uncertainty and complexity are the greatest challenges for projecting future heat-related mortality. The major difficulties are the long timescale over which climate change will occur; the diversity of potential impacts on health; and the complex interactions among demographic changes, socioeconomic development, technological innovation, and other environmental drivers (Ebi 2008; Frumkin and McMichael 2008; Gosling et al. 2008; Kinney et al. 2008). Currently, there are no guidelines for scenario-based projection research on heat-related mortality, and only a few studies have examined this issue. This article aims to fill this knowledge gap using a systematic review and to make recommendations for future research.

## Search Strategy and Selection Criteria

A literature search was conducted in August 2010 using the electronic databases PubMed (National Library of Medicine 2010), Scopus and ScienceDirect (Elsevier 2010), ProQuest (2010), and Web of Science (Thomson Reuters 2010). The search was limited to journal articles published in English from January 1980 through July 2010. The key words used were heat, temperature, mortality, death, climate change, projection, and scenario. References and citations of the articles identified were inspected to ensure that all relevant articles were included.

Three inclusion criteria were used to select articles. First, articles had to include at least one projection of future heat-related mortality; studies of the climate change impact solely on future infectious diseases or air pollution were excluded. Second, in order to obtain authoritative information, this review included only peer-reviewed journal articles; books, reports, and conference abstracts were excluded. Third, we included only quantitative, empirical studies; reviews and qualitative studies were excluded.

## Results

We found 14 studies, including three that considered both the future impacts of heat and air pollution on mortality. These studies are summarized in Table 1, with the most recent studies listed first.

**Table 1:** Characteristics of studies that projected heat-related mortality under climate change scenarios.

Reference	Setting	Study period	Mortality	Temperature exposure	Projection results
Jackson et al. 2010		Four areas in Washington State: the Greater Seattle Area, Tri-Cities, Spokane County, and Yakima County, USA		2025, 2045, 2085		Heat events and air pollution		Humidex		The largest number of projected deaths in all years and scenarios for the Seattle region was found for persons ≥ 65 years of age. Under the middle warming scenario, this age group is expected to have 96, 148, and 266 excess deaths in 2025, 2045, and 2085, respectively.
Hayhoe et al. 2010		Chicago, USA		1961–1990, 2010–2039, 2040–2069, 2070–2099		Heat related		Spatial Synoptic Classification		Annual average mortality rates by the end of this century are projected to equal 1995 levels under lower B1 emissions scenario and to reach twice 1995 levels under higher A1FI emissions scenario.
Baccini et al. 2010		Fifteen European cities: Athens, Barcelona, Budapest, Dublin, Helsinki, Ljubljana, London, Milan, Paris, Prague, Rome, Stockholm, Turin, Valencia, Zurich		2030		Heat related		Maximum apparent temperature		The number of heat-related deaths per summer ranged from 0 in Dublin to 423 in Paris. The highest impact was in three Mediterranean cities (Barcelona, Rome, and Valencia) and in two continental cities (Paris and Budapest). The largest impact was on persons > 75 years of age, but in some cities relatively large proportions of heat-related deaths were also found among younger adults.
Doherty et al. 2009		Fifteen U.K. conurbations in England and Wales		2003, 2005, 2006, 2030		Heat and ozone exposure		Mean temperature		In the summers of 2003, 2005, and 2006 around 5,000 deaths were attributable to heat in England and Wales. The authors did not present the 2030 projection results.
Cheng et al. 2009b		Four cities in south-central Canada: Montreal, Ottawa, Toronto, and Windsor		2040–2059, 2070–2089		Differential and combined impacts of extreme temperatures and air pollution		Synoptic weather typing		Heat-related mortality is projected to be more than double by the 2050s and triple by the 2080s from the current levels. Cold-related mortality could decrease by 45–60% and 60–70% by the 2050s and the 2080s, respectively. Population acclimatization to increased heat could reduce future heat-related mortality by 40%.
Gosling et al. 2009		Six U.S., European, and Australian cities: Boston, Budapest, Dallas, Lisbon, London, and Sydney		2070–2099		Summer heat related		Maximum temperature		Higher mortality is attributed to increases in the mean and variability of temperature with climate change rather than with the change in mean temperature alone. Acclimatization to an increase of 2°C reduced future heat-related mortality by approximately half that of no acclimatization in each city.
Doyon et al. 2008		Three cities in Québec, Canada: Montréal, Québec, and Saguenay		2020, 2050, 2080		Heat and cold related		Mean temperature		A significant increase in summer mortality is projected, and a smaller but significant decrease in fall. The slight changes in projected mortality for winter and spring were not statistically significant. The changes in projected annual mortality are dominated by an increase in mortality in summer, which is not balanced by the decrease in mortality in fall and winter. The difference between the mortality changes projected with the A2 or B2 scenarios was not statistically significant.
*continued next page*										
Table 1. *continued*										
Reference		Setting		Study period		Mortality		Temperature exposure		Projection results
Knowlton et al. 2007		New York City region, USA		1990s, 2050s		Summer heat related		Mean temperature		Projected increases in heat-related mortality by the 2050s ranged from 47% to 95%, with a mean 70% increase compared with the 1990s. Acclimatization reduced future summer heat-related mortality by about 25%. Urban counties had greater numbers of deaths and smaller percentage increases than did less urbanized counties.
Takahashi et al. 2007		The entire globe		2091–2100		Heat related		Maximum temperature		When the changes of excess mortality due to heat were examined by country, the results showed increases of approximately 100% to 1,000%. When considered with present population densities, significant increases in excess mortality are predicted in China, India, and Europe.
Hayhoe et al. 2004		Los Angeles, USA		2070–2099		Heat related		Maximum apparent temperature		From a baseline of around 165 excess deaths during the 1990s, heat-related mortality was projected to increase by about 2–3 times under B1 and by about 5–7 times under A1FI by the 2090s if acclimatization was taken into account. Without acclimatization, these estimates were 20–25% higher.
Dessai 2003		Lisbon, Portugal		2020s, 2050s, 2080s		Summer heat related		Maximum temperature		Annual heat-related mortality was estimated to increase from between 5.4–6.0 (per 100,000) for 1980–1998 to between 5.8–15.1 for the 2020s. By the 2050s, the potential increase ranged from 7.3 to 35.6. For the summer months mean approach, acclimatization reduced deaths on average by 15% for the 2020s and 40% for the 2050s, respectively, whereas for the 30-day running mean approach acclimatization reduced deaths by 32% and 54%.
Guest et al. 1999		Five cities in Australia: Adelaide, Brisbane, Melbourne, Perth, Sydney		2030		Heat and cold related		Temporal synoptic indices		After allowing for increases in population and combining all age groups, the projection is a 10% reduction in mortality in the year 2030 when considering reduced winter mortality.
Martens 1998		Twenty cities worldwide: Mauritius, Buenos Aires, Caracas, San Jose, Santiago, Beijing, Guangzhou, Singapore, Tokyo, Amsterdam, Athens, Budapest, London, Madrid, Zagreb, Los Angeles, New York, Toronto, Melbourne, Sydney		2040–2100		Heat and cold related		Mean temperature		For most of the cities, global climate change is likely to lead to a reduction in mortality rates because of decreasing winter mortality. This effect is most pronounced for cardiovascular mortality in elderly people in cities that experience temperate or cold climates at present.
Kalkstein and Greene 1997		Forty-four U.S. cities with > 1 million people		2020, 2050		Heat and cold related		Spatial Synoptic Classification		Increases in the frequency of summer high-risk air masses could contribute to significantly higher summer mortality, especially for the 2050 models. Increases in heat-related mortality ranged from 70% for the most conservative GCM to > 100% for the other GCMs in 2050, even if the population acclimatized to the increased heat. Winter mortality would drop slightly, but this would not offset the increases in summer mortality to any significant degree.

*Main findings.* In a study of the possible health impacts of climate change in 44 U.S. cities, Kalkstein and Greene (1997) estimated that increases in heat-related mortality would range from 70% to > 100% in 2050, relative to the baseline 1964–1991 summer mortality. Winter mortality would drop slightly, but this would not offset the increases in summer mortality to any significant degree. Large increases in heat-related mortality have also been projected for other cities in the United States. Heat-related mortality in Los Angeles (Hayhoe et al. 2004) by the 2090s was projected to increase by two to three times under the lower B1 emissions scenario and by five to seven times under the higher A1FI emissions scenario relative to 1961–1990. In the New York City metropolitan region, projected increases in heat-related mortality ranged from 47% to 95% by the 2050s, with a mean 70% increase compared with the 1990s (Knowlton et al. 2007). Annual average mortality rates in Chicago by the end of this century were projected to be equal to those of 1995-like heat waves under the B1 emissions scenario and to reach twice the 1995 levels under the A1FI emissions scenario (Hayhoe et al. 2010). In Washington State, the largest projected excess deaths for the Seattle region occurred in those ≥ 65 years of age. Holding the population projection constant at the 2025 level, and using a moderate warming scenario, this age group was projected to have 96, 148, and 266 excess deaths due to heat events in 2025, 2045, and 2085, respectively (Jackson et al. 2010).

In three cities in Canada, Doyon et al. (2008) projected a significant increase in temperature-related mortality in summer that was not offset by a significant but smaller estimated decrease in fall and winter mortality. Cheng et al. (2009b) projected that heat-related mortality in four Canadian cities would more than double by the 2050s and triple by the 2080s, and that cold-related mortality could decrease by 45–60% by the 2050s and by 60–70% by the 2080s.

In Australia, Guest et al. (1999) applied the climate–mortality relationship estimated for 1979–1990 to scenarios for climate and demographic changes to predict potential impacts on mortality in five major cities in 2030. They estimated that changes in mortality due to direct climatic effects would be small after considering reduced winter mortality. However, we feel that this is probably because of the relative insensitivity of warming to emissions scenarios, over the short period to 2030 (Murphy et al. 2009).

Baccini et al. (2010) projected that climate change in Europe would have the greatest impact on three Mediterranean cities (Barcelona, Rome, and Valencia) and two continental cities (Paris and Budapest). The largest estimated impact according to age was on persons > 75 years of age, but in some cities relatively large proportions of heat-related deaths were also projected for younger adults. Dessai (2003) projected that annual heat-related deaths in Lisbon would increase from 5.4–6.0 per 100,000 in 1980–1998 to 5.8–15.1 in the 2020s, and to 7.3–35.6 in the 2050s. Takahashi et al. (2007) projected global rates of excess deaths due to heat would increase by 100% to 1,000% in the 2090s relative to the 1990s. The authors projected that the burden of future heat-related mortality would vary by country, with greater effects in China, India, and Europe because of the high population density of these regions. Their estimates were based on World Bank population estimates for the year 2000 and assumed no change in future population density or age composition.

It has been estimated that population acclimatization could reduce future heat-related mortality by 20–25% in Los Angeles (Hayhoe et al. 2004), 25% in New York City (Knowlton et al. 2007), and 40% in south-central Canada (Cheng et al. 2009b). In Lisbon, acclimatization could reduce heat-related deaths on average by 15% in the 2020s and 40% in the 2050s, relative to projections assuming no acclimatization (Dessai 2003). Gosling et al. (2009) estimated that acclimatization to an extra 2°C in maximum temperature would reduce future heat-related mortality by 50%. However, complete acclimatization to high temperatures is unlikely because it would require extensive improvements to existing buildings (particularly in poor areas), and because the capacity for acclimatization will be reduced in vulnerable populations such as patients with advanced heart disease (Kalkstein and Greene 1997).

In summary, most projections showed that climate change would result in a substantial increase in heat-related mortality. Also, most studies did not consider demographic changes that are expected to result in an aging population (who are more susceptible to heat), which could lead to an underestimation of future heat-related mortality. Acclimatization could reduce future heat-related mortality, but it would not entirely eliminate the impacts of climate change on mortality. The studies identified by our review used many different climate models, emissions scenarios, time periods, temperature exposures, and assumptions. It is therefore difficult to compare the different studies using standardized results that are stratified by periods and locations. It is also not possible to conduct a meta-analysis to create a combined estimate of the impacts of climate change on heat-related mortality.

*Methodological issues.* Projecting heat-related mortality under climate change models and scenarios requires understanding of the historical temperature–mortality relationships and consideration of the future changes in climate, population, and acclimatization. Estimation of the possible health consequences of climate change is inherently difficult and involves numerous uncertainties, as outlined below and summarized in Table 2.

**Table 2:** Methodological issues of studies that projected heat-related mortality under climate change scenarios.

Reference	Baseline temperature–mortality relationship	Climate change scenario*a*	Climate model*b*	Downscaling	Demographic change	Acclimatization
Jackson et al. 2010		The relationship between age- and cause-specific mortality from 1980 to 2006 and heat events at the 99th Humidex percentile from 1970 to 2006		Three scenarios: high, moderate, and low summer warming		The high scenario was the HadCM-A1B model, the low scenario was the PCM1-B1 model, and the middle scenario was the mean of the two composite models using either the A1B or B1 emissions scenario		Did not consider		Projected county population estimates by age group were obtained for the years 2005–2030; in predicting future heat-related mortality, the population was held constant at the 2025 projection, which allowed differences in excess deaths between years to be interpreted as the component due to climate change		Assumed no acclimatization
Hayhoe et al. 2010		The Spatial Synoptic Classification method to quantify the meteorological and seasonal contributions to heat-related mortality in Chicago for the years 1961–1990		Two scenarios: A1FI and B1		Three GCMs: GFDL CM2.1, HadCM3, and PCM		Statistical downscaling		Assumed no demographic change		Assumed no acclimatization
Baccini et al. 2010		The study of city-specific air-quality–adjusted estimates of mortality risk by maximum apparent temperature over the years 1990–2001 (Baccini et al. 2008)		Three scenarios: B1, A1B, and A2		Did not use any climate models; B1 equal to 1.8°C, A1B equal to 2.8°C, A2 equal to 3.4°C increase in temperature by 2090–2099 relative to 1980–1999		Did not consider		Assumed no demographic change		Assumed no acclimatization
Doherty et al. 2009		Estimates of ozone- and heat-mortality were based on time series of daily mortality for the period May–September 1993–2003 for the 15 English and Welsh conurbations		Three scenarios: optimistic (maximum feasible reduction), pessimistic (SRES A2), and current legislation		The coupled WRF-EMEP4UK model was used to simulate daily surface temperature and ozone		Did not consider		Assumed no demographic change		Did not consider
Cheng et al. 2009b		An automated synoptic weather typing approach was used to assess the relationship between weather types and elevated mortality for the years 1954–2000 (Cheng et al. 2009a)		Three scenarios: IS92a, A2, and B2		Canadian GCM CGCM1, Canadian GCM CGCM2, U.S. GCM GFDL-R30		Statistical downscaling		Assumed no demographic change		The five hottest and coolest summers in each city were selected; the difference in daily mean deaths between the hottest and coolest summers was assumed to be due to acclimatization
Gosling et al. 2009		The relationship between temperature and summer mortality in different cities for the historical years (Boston 1975–1998, Budapest 1970–2000, Dallas 1975–1998, Lisbon 1980–1998, London 1976–2003, and Sydney 1988–2003) (Gosling et al. 2007)		Two scenarios: A2 and B2		The U.K. HadCM3 GCM		Did not consider		Assumed no demographic change		Three possibilities: no acclimatization, acclimatization to an increase of 2°C, and acclimatization to an increase of 4°C relative to present
Doyon et al. 2008		The relationship between total mortality (excluding trauma) and climate for different cities during the period 1981–1999		Two scenarios: A2 and B2		The U.K. HadCM3 GCM		Statistical downscaling		Assumed no demographic change		Assumed no acclimatization
Knowlton et al. 2007		Derived from a study of observed temperature and mortality in 11 eastern U.S. cities for the years 1973–1994 (Curriero et al. 2002)		Two scenarios: A2 and B2		The GISS–MM5 linked model		Dynamic downscaling		Assumed no demographic change		Modeled acclimatization by using a heat exposure–mortality response function derived from two U.S. cities with current observed temperatures similar to those projected for the 2050s in the New York region
*continued next page*												
Table 2. *continued*												
Reference		Baseline temperature–mortality relationship		Climate change scenario*a*		Climate model*b*		Downscaling		Demographic change		Acclimatization
Takahashi et al. 2007		The relationship between temperature and mortality in the 47 prefectures of Japan for the years 1972–1995		One scenario: A1B		CCSR/NIES/FRCGC GCM		Did not consider		Assumed no demographic change		Assumed no acclimatization
Hayhoe et al. 2004		An algorithm was developed for all days with maximum apparent temperatures at or above 34°C to estimate daily heat-related mortality during 1961–1990		Two scenarios: B1 and A1FI		Two GCMs: PCM and HadCM3		Statistical downscaling		Assumed no demographic change		Selected “analogue summers” best duplicating the summers as expressed in the climate change scenarios; for Los Angeles, the five hottest summers over the past 24 years were selected based on mean summer apparent temperature values
Dessai 2003		The climate–mortality relationship of the summer months of 1980–1998 (Dessai 2002)		The median of the modeled values was used because at the time RCMs had not been run with all the SRES scenarios		Two RCMs: PROMES and HadRM2		High-resolution RCMs that yield greater spatial details about climate		The population growth rate from each SRES storyline (A1, A2, B1, and B2) was applied to the 1990 Lisbon population to produce 10-year spaced population figures until 2100; the median population from these calculations was used for simplicity		Assumed that complete acclimatization to an extra 1°C (compared with the 1990s) is reached after three decades
Guest et al. 1999		The relationship between temperature and mortality (cause specific and all cause) during the period 1979–1990		Low and high climate change scenarios		The CSIRO-Mk2 GCM		Did not consider		Data on projected population for 2030 were obtained; these data account for aging as well as growth of the population		Assumed no acclimatization
Martens 1998		A meta-analysis giving an aggregated effect of mean temperature on mortality for total, cardiovascular, and respiratory mortality		Three GCMs scenarios		Three GCMs: ECHAM1-A, UKTR, and GFDL89		Did not consider		Assumed no demographic change		The sensitivity of heat- and cold-related mortality to physiological as well as socioeconomic adaptation was examined
Kalkstein and Greene 1997		An air mass–based synoptic procedure was used to evaluate the relationships between synoptic events and mortality for the period of 1964–1991		Three GCMs scenarios		Three GCMs: GFDL, UKMO, and the Max Planck Institute for Meteorology model		Did not consider		Assumed no demographic change		Analogue cities were established for each city; these analogues represent cities whose present climate approximates the estimated climate of a target city as expressed by the GCMs
Abbreviations: GCM, general circulation model; RCM, regional climate model. For details on the different climate models and emissions scenarios, see IPCC 2007b. **a**Climate change scenario is a coherent description of the change in climate under specific assumptions about the growth of greenhouse gas emissions and about other factors that may influence future climate. **b**Climate model is mathematical description of the climate system that enables us to project how the climate may change in the future.												

*Baseline temperature–mortality relationships.* Mortality projections are based on historical exposure–response functions of temperature and mortality that are applied to climate change models and emissions scenarios to estimate future heat-related mortality. Therefore, it is important to consider which measure of temperature is the best predictor of mortality.

Maximum temperature and mean temperature are commonly used measures of heat exposure. For example, Dessai (2002) modeled the relationship between maximum temperature and excess deaths in Lisbon during the summer months in 1980–1998 and then applied different climate and population change scenarios to the model to assess potential impacts on mortality in the 2020s and 2050s (Dessai 2003). Knowlton et al. (2007) projected the impacts of climate change on summer mortality using modeled daily mean temperatures for New York City. Martens (1998) reviewed the literature on the relationship between mean temperature and mortality and derived a pooled estimate of the effect of temperature on mortality using meta-analysis.

Others have used composite indices, which examine the combined effects of ambient temperature, humidity, and other meteorological variables. For example, the synoptic approach is an air-mass–based method that quantifies the effect of air and dew point temperatures, wind speed, cloud cover, barometric pressure, and others. The apparent temperature and Humidex combine the effects of temperature and humidity. Kalkstein and Greene (1997) used the Spatial Synoptic Classification to evaluate climate–mortality relationships in the U.S. cities. Guest et al. (1999) described the climate–mortality relationships by temporal synoptic indices in Australia. Hayhoe et al. (2004) identified maximum apparent temperature thresholds that were associated with rising heat-related mortality in California. Jackson et al. (2010) examined the historical relationship between age- and cause-specific mortality rates and heat events at the 99th Humidex percentile in Washington State.

Barnett et al. (2010) examined which measure of temperature is the best predictor of mortality. The authors compared seven temperature measures: maximum, mean, and minimum temperature; maximum, mean, and minimum apparent temperature; and the Humidex, based on daily data for 107 U.S. cities during 1987–2000. No one temperature measure was superior to the others. The strong correlation between different temperature measures suggests that they have a similar predictive ability. For the projection research of heat-related morality, we therefore propose that the temperature measure can be chosen based on practical concerns, such as using mean temperature, which may be commonly available from the climate models.

The choice of model is probably a more important consideration than the choice of temperature measure. Hajat et al. (2010b) compared the predictive capacity of four approaches for identifying dangerous hot days: physiological classification, synoptic approach, temperature–humidity index, and temperature–mortality relationship. There was little agreement across the different approaches, but in general, modeling the temperature–mortality relationship most accurately identified days of highest excess mortality. For projection research, we recommend examining the heat threshold and slope using a temperature–mortality relationship based on a continuous scale of temperature values.

**Figure 1:**
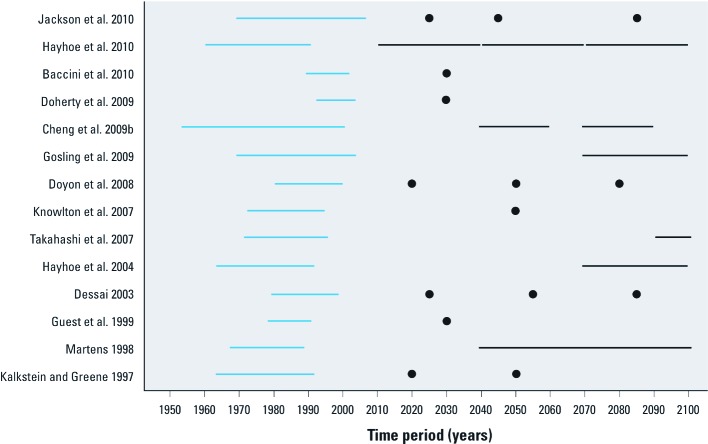
Time periods used by studies of climate change and projected mortality, ordered by date of publication. Blue lines show the baseline time periods; black lines or black circles show the projection time periods.

Choosing a baseline time period for the temperature–mortality relationship is also important. Temperature–mortality relationships in the same city can be very different between the 1960s and the 2000s. Differences could be due to socioeconomic development, demographic change, and population acclimatization. Differences in the time periods used to estimate the historical temperature–mortality relationships also make it difficult to compare projections across studies. Figure 1 highlights the variability of different time periods used in each study. Because daily mortality data often are not available before 1990 in many cities, the time period 1996–2005, which centered on 2000, is recommended as the baseline.

*Climate change projections.* Another fundamental issue for projecting heat-related mortality is the modeling of future climate. The IPCC has defined a set of 40 SRES scenarios that covered a wide range of the main driving forces of future greenhouse gas emissions (IPCC 2007b). These scenarios are structured in four major families labeled A1, A2, B1, and B2. A1 represents rapid economic growth, global population peaking in midcentury, and rapid introduction of new and efficient technologies. A1 has three subgroups: A1FI (fossil intensive), A1T (nonfossil), and A1B (balanced). A2 represents high population growth, slow economic development, and slow technological change. B1 represents the same population growth as A1 but rapid changes in economic structures toward a service and information economy. B2 represents intermediate population and economic growth with local solutions to economic, social, and environmental sustainability. The emissions scenarios can be used to project future climates based on various general circulation models (GCMs) (IPCC 2007b).

Selecting climate models is also not a trivial task, given the strengths and weaknesses of various GCMs. There are different types of GCMs, depending on whether they incorporate dynamics from the atmosphere, the ocean, or both (IPCC 2007b). However, although all GCMs attempt to accurately represent climate processes, this gives rise to different GCMs adopting different representations and hence generates different climate change projections, even when assuming the same pathway of future emissions (IPCC 2007b). For more detailed information on emissions scenarios and GCMs, see IPCC (2007b).

In the existing studies, Knowlton et al. (2007) considered two of the emissions scenarios, A2 and B2, which assume relatively high and low future emissions, respectively. They used both scenarios to model daily mean temperatures in the 1990s and 2050s. Gosling et al. (2009) applied the same scenarios to model daily maximum temperatures during 1961–1990 and 2070–2099. Hayhoe et al. (2004) projected future climates based on the higher A1FI and lower B1 emissions scenarios. Jackson et al. (2010) selected three climate change scenarios for high (A1B), low (B1), and moderate (A1B and B1 combined) summer warming.

Adding to the uncertainties of emissions scenarios, different GCMs were often used to simulate the future or current climates. Kalkstein and Greene (1997) used three GCMs in their study: the GFDL model, the UKMO model, and the Max Planck model. Guest et al. (1999) used the CSIRO-Mk2 to project regional monthly mean changes in temperature, rainfall, and other climates. Also, because the spatial resolution of GCM results is too coarse to be used directly in the impact assessment at a local scale, downscaling methods were introduced. Cheng et al. (2009b) used a statistical downscaling approach to downscale daily outputs from five GCMs for selected cities. Knowlton et al. (2007) used the dynamic downscaling approach, in which the GCM outputs were used as initial and boundary conditions for finer-scale simulations by the climate model. Daily climate projections from regional climate models (RCMs) were also applied to climate–mortality studies. RCMs are higher-resolution climate models that can be nested within GCMs to provide more detailed simulations for a particular area. For example, Dessai (2003) used results from two RCMs (PROMES and HadRM2) that yield greater spatial details about climate.

Climate change scenarios will determine the size of the predicted future heat-related mortality. Therefore, it is important to consider different emissions scenarios in the impact assessment, offering a range of possible future climates and health impacts. The uncertainty associated with future emissions has been recognized in the U.K. climate projections (UKCP09) by giving probabilistic projections that correspond to each of the three different emissions scenarios: high, medium, and low (Murphy et al. 2009). These scenarios correspond to three of the commonly used emissions scenarios in SRES: A1FI, A1B, and B1, respectively.

Because of the varying sets of strengths and weaknesses of different GCMs, the IPCC suggested that no single GCM can be considered the best and that multiple GCMs should be used to account for modeling uncertainties (IPCC 2007b). Also, climate projection data at a higher spatial resolution will be more valuable, especially for information on the urban heat island effect. Better information on the probability of heat waves occurrences will also increase the accuracy of projections concerning the health impacts of climate change (Gawith et al. 2009).

*Demographic changes.* Challenges also arise from the uncertainties of future demographic changes that will modify the future sensitivity of populations to heat stress. Growing numbers of older adults will increase the proportion of the population at risk (Kovats and Hajat 2008; Luber and McGeehin 2008; O’Neill and Ebi 2009). In addition to having a diminished physiological ability to cope with heat, the elderly are more likely to live alone, have reduced social contacts, and experience poor health (Hajat et al. 2010a). Also, the effects of heat on mortality appear sometimes to be greater in women, especially elderly women (Ishigami et al. 2008; Vaneckova et al. 2008a).

To project the effects of climate change independent of effects of population trends, one approach is to assume that the population size and age structure will remain constant. For example, Knowlton et al. (2007) assumed that population totals for each of the 31 counties in New York City, based on data obtained from the U.S. Census 2000 survey, were held constant throughout the modeling period. Baseline mortality rates for all age groups were also held constant. Similarly, Cheng et al. (2009b), Gosling et al. (2009), Takahashi et al. (2007), and Hayhoe et al. (2004) did not account for population changes.

If susceptible populations are considered, then future demographic trends should be addressed. Guest et al. (1999) used data on the projected population for 2030 that accounted for an aging population. Jackson et al. (2010) obtained the county population estimates by age group for the years 2005–2030. The population was held constant for the 2025 projection, allowing differences in excess deaths between years to be interpreted as the component due to climate change. Dessai (2003) estimated population scenarios for Lisbon in line with the SRES. The population growth rates from each SRES storyline were applied to the 1990 Lisbon population to produce future population figures until 2100, and the median population from these calculations was used. National population projections were not used because they did not go far enough into the future.

*Population acclimatization.* How populations may acclimatize to elevated temperatures over time is another issue affecting mortality projections (Kalkstein and Greene 1997). Acclimatization can be a physiological process of humans adjusting to changes in their environment (Moseley 1994). People may also adapt to extreme heat through increased use of air conditioning, modified behavior patterns, and improved building designs and urban planning (Gosling et al. 2008; Kinney et al. 2008; O’Neill et al. 2005).

One approach is to assume that no acclimatization takes place in the future. For example, Baccini et al. (2010) argued that epidemiological evidence of the extent to which short- or long-term acclimatization alters mortality risk is limited and sometimes discordant. For their projections the authors therefore assumed that no acclimatization occurred, and hence there would be no future change in the temperature–mortality relationship. Jackson et al. (2010), Hayhoe et al. (2010), Doyon et al. (2008), Takahashi et al. (2007), Guest et al. (1999), and Martens (1998) also assumed no future acclimatization.

To incorporate acclimatization, one approach is to use the exposure–response curves from analogue cities. These analogues represent cities whose present climate best approximates the estimated future climate of a target city. For example, Knowlton et al. (2007) modeled acclimatization in New York City using a temperature–mortality response function derived for Washington, DC, and Atlanta, Georgia (USA), which had mean summer temperatures for 1973–1994 that were within approximately 1°F of projected temperatures for the New York City region in the 2050s. However, estimates based on this approach may be biased if social, economic, and demographic characteristics related to mortality differ greatly between the target and analogue regions.

Another approach involves the use of analogue summers from the same city to model population acclimatization. Hayhoe et al. (2004) used analogue summers whereby future acclimatization was based on the temperature–mortality relationship only in the hottest summers on record. Cheng et al. (2009b) identified the five hottest and five coolest summers during 1953–2000 and attributed the differences in daily mean deaths between the hottest and coolest summers to acclimatization.

Others have accounted for acclimatization by shifting current temperature–mortality relationships to the future. Using this method, the heat threshold increases with time but the slope of the temperature–mortality relationship remains unchanged. Dessai (2003) assumed that complete acclimatization to an extra 1°C warming in maximum temperature is reached every three decades. Gosling et al. (2009) considered three possibilities of future acclimatization: no acclimatization, acclimatization to an increase of 2°C, and acclimatization to an increase of 4°C. How acclimatization might reduce the impacts of climate change is not well understood, and there is no consensus on how to estimate its effect. We recommend conducting sensitivity analyses using different approaches to model population acclimatization when projecting future heat-related mortality.

## Discussion

The potential impacts of climate change on heat-related mortality are the subject of increasing public health concern (IPCC 2007a; WHO 2008). A variety of methods have been used to project future heat-related mortality. Although each of the methods has limitations, collectively they provide insight concerning projections of heat-related mortality under climate change scenarios. Projecting heat-related mortality under a changing climate requires analysis of historical exposure–response functions of temperature and mortality and consideration of the future changes in climate, population, and acclimatization.

Reliable climate projections are now increasingly available for many regions of the world because of advances in climate modeling (IPCC 2007b). The GCMs provide credible estimates of future climate change. Their credibility comes from the well-established physical basis of climate models, from the ability to simulate important aspects of the current climate, and from the ability to reproduce features of past climate changes (IPCC 2007b). Using the multimodel ensembles, along with statistical and dynamical techniques for regionalizing GCM outputs, climate researchers have moved toward representing changes in future climate with probabilities (IPCC 2007b; Murphy et al. 2009). It is possible that regional climate projections for a given emissions scenario could soon become routine. There is an urgent need for the environmental health community to conduct evidence-based assessments of the health impacts of climate change by closer collaboration with climate researchers.

Uncertainties in climate change projections should also be considered. Uncertainties may arise from model parameters, or from structural uncertainties as some processes in the climate system are not fully understood or are impossible to resolve because of computational constraints (IPCC 2007b). Thus, the estimates of future heat-related mortality contain uncertainties that need to be carefully interpreted for policy implications.

Another important consideration is the stability of temperature–mortality relationships over time. There are limitations to using present-day exposure–response functions to project future heat-related mortality levels, because the relationship between heat and mortality may change over time. Changes in mortality risk may occur because of an aging population or because of acclimatization, socioeconomic development, and adaptation strategies (Ebi 2008; Gosling et al. 2009). If we do not consider adaptations in the modeling, it would likely lead to an overestimate of the future effects of heat. For instance, adaptation may occur through improved building design and better city planning and land-use patterns, such as green roofs, reflective surfaces on roads and buildings, tree planting, and preservation of regional green space (Luber and McGeehin 2008; Younger et al. 2008). Adaptation may include changes in exposure patterns as people may spend less time outside, thereby altering the impact of heat (Kinney et al. 2008). The prevalence of air conditioning has increased, and this trend is expected to continue (O’Neill et al. 2005). The implementation of heat health warning systems is becoming more widespread, and these systems may reduce the health risks from heat waves (Ebi and Schmier 2005).

There is conflicting evidence of air pollution being a confounder and an effect modifier of the temperature–mortality relationship. Some studies reported confounding effects of air pollutants on the association between temperature and mortality (Medina-Ramon and Schwartz 2007; Ren et al. 2006), whereas others found no evidence of confounding or effect modification (Basu et al. 2008; Zanobetti and Schwartz 2008). Recent evidence has indicated that the confounding effect of air pollution is relatively small, and there are independent effects of air pollution and temperature on mortality (Basu 2009). However, because extreme heat events and increased levels of air pollution (e.g., ozone) often coincide, it is necessary to understand not only the independent effects of heat on mortality but also any combined effects of heat and air pollution (WHO 2009a). Air pollution is expected to increase in urban areas due to climate change, so the joint exposure of urban populations to high temperatures and air pollutants will increase in the future (O’Neill and Ebi 2009). Future heat-related mortality may also be due to indirect causes, such as deaths due to increased ozone caused by increased temperature, or synergistic effects of heat and air pollution (Barnett and Hansen 2009).

Short-term mortality displacement or “harvesting” is another important issue. Mortality displacement suggests that some heat-related deaths in already frail populations are only hastened by heat exposure (Kovats and Hajat 2008). If most heat-related deaths were in the very elderly who had only a life expectancy in single years, the public health significance of heat-related deaths would be reduced. Few studies have investigated the degree of mortality displacement for heat-related deaths (Hajat et al. 2005; Toulemon and Barbieri 2008). The accurate estimation of years of life lost because of high temperatures remains unknown and would likely vary as a function of the severity and duration of heat events (Kinney et al. 2008).

As global average temperatures increase, heat-related mortality will increase, but overall effects on mortality could be offset somewhat by reductions in cold-related mortality (Guest et al. 1999; Martens 1998). For example, Davis et al. (2004) estimated that a uniform 1°C warming results in a net mortality decline of 2.65 deaths per standard million per metropolitan areas, with 3.61 additional deaths in summer and 8.92 fewer deaths in winter in U.S. cities. Nevertheless, many scientists believe that the future increase in heat-related mortality is unlikely to be offset by the reduction in cold-related mortality, especially in the medium to long term (Costello et al. 2009; McMichael et al. 2003; Patz et al. 2005; WHO 2008). More studies are needed to understand how the balance of heat-related and cold-related mortality could change under different climate change and socioeconomic scenarios (IPCC 2007a). However, because public health adaptation strategies could be different for heat waves and cold spells, it is better to separate the projections of heat- and cold-related mortality rather than presenting only a net mortality change (Gosling et al. 2008).

Although the magnitude of future climate change remains uncertain, climate modeling exercises indicate that future heat waves will be more frequent, more intense, and longer lasting (Fischer and Schär 2010; Kintisch 2009; Meehl and Tebaldi 2004). Efforts to better understand how climate change will affect population health, especially among the most vulnerable groups, are necessary (Ebi 2008; WHO 2009b). Given uncertainties in our understanding of the future population vulnerability to heat, it is important to use various methods to capture a plausible range of the health impacts of climate change (Kinney et al. 2008). Further research is needed to provide a stronger theoretical framework for projecting heat-related mortality under climate change scenarios, including better understanding of socioeconomic development, adaptation strategies, land-use patterns, air pollution, and mortality displacement.

## Conclusions

Climate change is likely to cause increased heat-related mortality. A few studies have projected heat-related mortality under different climate change scenarios. Significant differences in projected mortality can be found in different emissions scenarios, suggesting that greenhouse gas mitigation policies are important for protecting human health. Although the methods used for projections are still in their early stages and have limitations, the need for evidence-based assessments of future health impacts of climate change is urgent. Such research will significantly contribute to assessing and managing the potential impacts of climate change on heat-related mortality.
